# Detection of *Streptococcus pneumoniae* from culture-negative dried blood spots by real-time PCR in Nigerian children with acute febrile illness

**DOI:** 10.1186/s13104-018-3770-2

**Published:** 2018-09-10

**Authors:** Pui-Ying Iroh Tam, Nelmary Hernandez-Alvarado, Mark R. Schleiss, Amy J. Yi, Fatimah Hassan-Hanga, Chuma Onuchukwu, Dominic Umoru, Stephen K. Obaro

**Affiliations:** 1 Division of Pediatric Infectious Diseases and Immunology, University of Minnesota, Minneapolis, MN USA; 2 Aminu Kano Teaching Hospital, Kano, Nigeria; 3 Federal Medical Center, Keffi, Nasarawa Nigeria; 4 Nyanya General Hospital, Abuja, Nigeria; 5 University of Nebraska Medical Center, Omaha, NE USA; 6 International Foundation Against Infectious Diseases in Nigeria, Abuja, Nigeria; 7 University of Abuja Teaching Hospital, Gwagwalada, Nigeria; 8 Malawi-Liverpool Wellcome Trust Clinical Research Programme, Blantyre, Malawi; 9 Liverpool School of Tropical Medicine, Liverpool, UK

**Keywords:** Bloodstream infections, Children, Dried blood spot, Febrile illness, Molecular diagnostics, Pneumococcus

## Abstract

**Objectives:**

Acute febrile illness is a common cause of hospital admission, and its associated infectious causes, of which a key bacterial causative agent is *Streptococcus pneumoniae*, contribute to substantial morbidity and mortality. We sought to evaluate the utility of real-time (rt)-PCR on dried blood spots (DBS) for diagnosis of *S. pneumoniae* in acute febrile illness among children presenting to hospitals in Nigeria. We previously described preliminary results in a sample of 537 patients. Here we present data from a larger collection of 1038 patients.

**Results:**

Using rt-PCR for *Streptococcus pneumoniae* on 1038 dried blood spots from children prospectively enrolled with acute febrile illness, including 79 healthy controls, we detected pneumococcal DNA in nine of 15 blood culture-positive specimens, one culture-negative specimen from a high-risk group, a culture-confirmed non-pneumococcal specimen and a healthy control. Six culture-positive isolates (40%) were negative. Sensitivity was 60%, specificity 99.7%, positive predictive value 75% and negative predictive value 99.4%. Rt-PCR of DBS has limited sensitivity in blood specimens from acute febrile illness in children.

## Introduction

Acute febrile illness is a common cause of hospital admission, and its associated infectious causes contribute to substantial morbidity and mortality worldwide. A key bacterial causative agent is *Streptococcus pneumoniae*, and in Africa rates of invasive pneumococcal disease (IPD) are estimated to be the highest of any continent, with recent pooled incidence rates of 62.6 per 100,000 person-years for children ≤ 5 years of age [1]. The lack of robust surveillance systems in low- and middle-income countries (LMICs) has made it challenging to diagnose the etiology of disease in acute febrile illness. Dried blood spots (DBS) hold promise as a method for surveillance of pathogens in LMICs, due to the low blood volumes involved, low cost, and ability for storage and transport at ambient temperature. We have previously conducted proof-of-concept studies and demonstrated the high detection rate of pneumococcal DNA in spiked samples on filter paper by real-time (rt)-PCR, and then evaluated clinical specimens with blood culture data. Using rt-PCR for pneumococcal DNA on DBS, we documented an overall sensitivity of 60% (95% CI 32.3–83.7%) and specificity of 99.4% (95% CI 98.3–99.9%) [2]. We suspected that low rates of pneumococcal detection by culture may be due to widespread non-prescription antimicrobial use prior to presentation for hospital care. We hypothesized that rt-PCR would identify pneumococcal DNA even in culture-negative subjects pretreated with antimicrobials. To demonstrate further proof of principle of the utility of real-time (rt)-PCR on dried blood spots (DBS) for diagnosis of *S. pneumoniae* in acute febrile illness, for this study we expanded our evaluation to include a larger collection of culture-negative specimens among children presenting to hospitals in Nigeria.

## Main text

### Materials and methods

Between September 2011–June 2015, children ≤ 5 years of age who presented to six hospital study sites in Abuja and Kano, central and northern Nigeria, respectively, with an acute febrile illness (temperature > 38.5 °C) associated with difficulty breathing or altered consciousness (as subjectively assessed by the clinician of record) were prospectively enrolled. Detailed description of these clinical sites have been previously described [3]. A variety of clinical diagnoses were included, and a sample of non-matched healthy infant controls was also enrolled. After informed consent was obtained, clinical history and physical examination findings were collected on a detailed questionnaire by the physician. Blood (1–3 mL) was obtained and then processed within 4 h for culture using the automated BACTEC incubator system (Becton–Dickinson, Temse, Belgium) and also spotted onto Whatman 903 or FTA filter paper (GE Healthcare BioSciences, Pittsburgh, PA). Healthy controls only had DBS obtained. DBS were stored in a − 80 °C freezer before transport to the University of Minnesota for processing. Using methods described and validated previously [2], DBS DNA extractions were used as a template in rt-PCR assays targeting *lytA* gene [4], and RNase P amplification was used as a control to confirm recovery of human cellular DNA. Positive results were defined as those with a cycle threshold (Ct) < 40. Values ≥ 40 were considered equivocal and were repeated using DNA extracted from the same DBS specimen. DBS samples were run in duplicate, and were counted as positive when both replicates tested positive.

This study was approved by the Ethics Committees of the Federal Capital Territory, Federal Medical Center Keffi, and Aminu Kano Teaching Hospital, and by the University of Nebraska Medical Center and University of Minnesota Institutional Review Boards. Written signed informed consent was obtained from the parent or guardian.

### Results

**Fig. 1:**
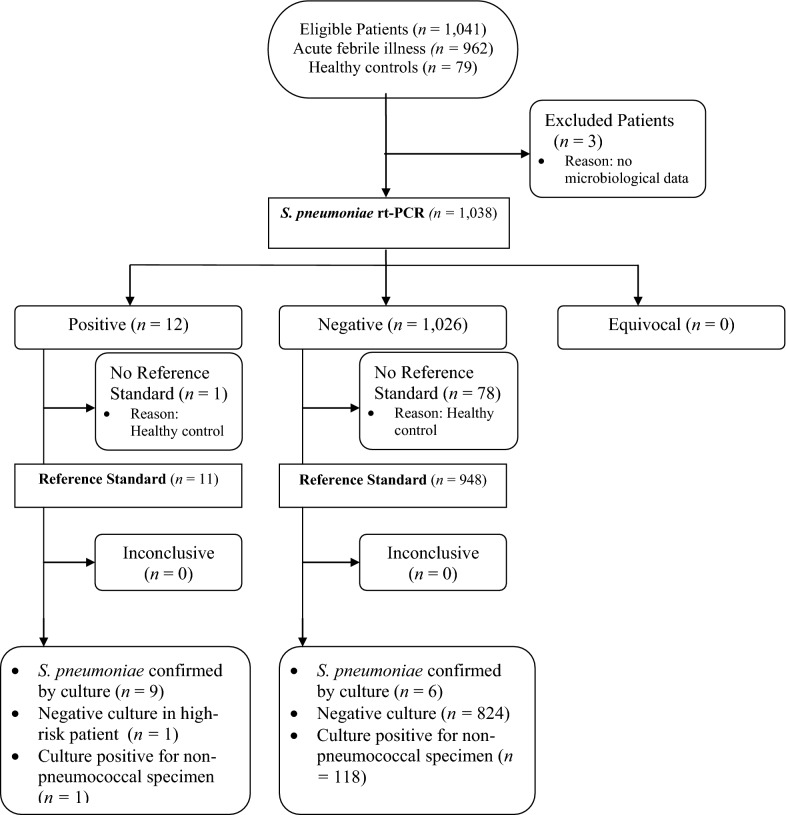
Standards for reporting of diagnostic accuracy studies (STARD) flowchart for comparative detection of *S. pneumoniae* DBS by rt-PCR with culture

Microbiological data were available for a total of 1038 specimens, excluding 79 healthy controls (7.6%). Among children with acute febrile illness, 825 (79.4%) blood cultures had no growth, 15 (1.4%) grew *S. pneumoniae*, 119 (11.5%) were positive for other bacteria, including 16 (13.4%) *Salmonella* Typhi, 6 (5.0%) *Salmonella* Group B, 5 (4.2%) *Haemophilus influenzae* type b, and 63 (52.9%) contaminants. Using rt-PCR, *S. pneumoniae* was detected from DBS in 9 culture-positive specimens, in a case of culture-confirmed non-pneumococcal bacteremia (*Haemophilus influenzae*) and in one healthy control (Fig. 1). Six culture-positive *S. pneumoniae* isolates (40%) were negative for pneumococcal DNA by rt-PCR. Of the specimens where there was no growth on blood culture, *S. pneumoniae* was detected from DBS by rt-PCR in one culture-negative specimen from a child with a clinical diagnosis of sepsis. Using culture as the gold standard, rt-PCR on DBS for *S. pneumoniae* has a sensitivity of 60%, specificity 99.7%, a positive predictive value of 75% and a negative predictive value of 99.4% (Table 1). For evaluation of culture-negative DBS, using PCR as the gold standard, culture for *S. pneumoniae* has a sensitivity of 75%, specificity 99.4%, positive predictive value of 60%, and negative predictive value of 99.7%.

**Table 1:** Performance of PCR on DBS for *Streptococcus pneumoniae* in acute febrile illness compared to gold standard of culture

	*S. pneumoniae* culture	
	Positive	Negative
*S. pneumoniae* PCR		
Positive	9	3
Negative	6	1020

### Discussion

There are several notable findings in our study of rt-PCR for pneumococcus on DBS of children with acute febrile illness, which includes a large collection of culture-negative specimens, that builds on what has been reported previously. Firstly, in a country where pre-vaccination studies indicate high rates of pneumococcal nasopharyngeal carriage [5], and where pneumococcal conjugate vaccine has been introduced but not yet widely distributed, few children with acute febrile illness are culture-positive for *S. pneumoniae.* Secondly, PCR missed over a third of *S. pneumoniae* culture-positive isolates. Thirdly, despite evaluating a larger collection of culture-negative specimens of children with acute febrile illness, *S. pneumoniae* was detected by rt-PCR in only one culture-negative DBS in a high-risk patient.

In this study, the culture-negative specimens possibly correlated with low bacterial density, making detection, even by molecular methods, difficult. The low sensitivity of molecular detection in DBS compared to blood culture may be due to use of frozen versus ambient specimens, low blood sample volume, extraction of DNA from the DBS, or the PCR assay itself. Earlier studies of PCR on DBS in children with a clinical diagnosis of pneumonia demonstrated a significant increase in identification of *S. pneumoniae* by PCR compared to blood bacterial culture [6]. However, background levels of PCR-positivity in healthy controls compared to pneumonia cases were noted in later studies [4], highlighting the poor specificity of pneumococcal PCR. In the Pneumonia Etiology Research for Child Health (PERCH) study, a significant degree of overlap existed among *S. pneumoniae* blood PCR-positive cases (5.5–11.5%) and controls (5.3–10.2%) in children from African countries [4]. Furthermore, cases confirmed to have non-pneumococcal DNA still had high PCR positivity rates for *S. pneumoniae* (11.2%), and was in fact higher than for cases with no confirmed bacterial pathogen (6.3%). This contrasted with microbiologically-confirmed pneumococcal pneumonia, which had disappointingly low blood PCR positivity rates (64.3%) [4].

## Limitations

This study on acute febrile illness included a large pediatric sample size where specimens were collected prospectively from several sites throughout northern and central Nigeria. There are some potential limitations. Even though filter paper can be stored at ambient temperature, prolonged storage can affect the DNA content [7]. Freeze–thaw cycles can also affect DNA integrity, although DNA from archival DBS has been shown to be stable after undergoing multiple freeze–thaw cycles [8]. The findings are generalizable to other pediatric centers in this region, but further studies are required to assess if the findings can be applied to other populations and settings.

In conclusion, the utility of rt-PCR for the detection of *Streptococcus pneumoniae* on culture-negative DBS in children with acute febrile illness is limited. Given the observations from this study as well as others such as PERCH, new diagnostic approaches beyond pneumococcal PCR protocols may need to be advocated to optimize ascertainment of disease burden in LMICs.

## Abbreviations

DBS: dried blood spots
IPD: invasive pneumococcal disease
LMIC: low- and middle-income country

## References

[1] Iroh Tam PY, Thielen BK, Obaro SK (2017). Childhood pneumococcal disease in Africa—a systematic review and meta-analysis of incidence, serotype distribution, and antimicrobial susceptibility. Vaccine.

[2] Iroh Tam PY, Hernandez-Alvarado N, Schleiss MR (2016). Molecular detection of *Streptococcus pneumoniae* on dried blood spots from febrile Nigerian children compared to culture. PLoS ONE.

[3] Obaro S, Lawson L, Essen U (2011). Community acquired bacteremia in young children from central Nigeria—a pilot study. BMC Infect Dis.

[4] Morpeth SC, Deloria Knoll M, Scott JAG (2017). Detection of pneumococcal DNA in blood by polymerase chain reaction for diagnosing pneumococcal pneumonia in young children from low- and middle-income countries. Clin Infect Dis.

[5] Adetifa IM, Antonio M, Okoromah CA (2012). Pre-vaccination nasopharyngeal pneumococcal carriage in a Nigerian population: epidemiology and population biology. PLoS ONE.

[6] Selva L, Benmessaoud R, Lanaspa M (2013). Detection of *Streptococcus pneumoniae* and Haemophilus influenzae type B by real-time PCR from dried blood spot samples among children with pneumonia: a useful approach for developing countries. PLoS ONE.

[7] Hwang J, Jaroensuk J, Leimanis ML (2012). Long-term storage limits PCR-based analyses of malaria parasites in archival dried blood spots. Malar J.

[8] Schwartz A, Baidjoe A, Rosenthal PJ, Dorsey G, Bousema T, Greenhouse B (2015). The effect of storage and extraction methods on amplification of *Plasmodium falciparum* DNA from dried blood spots. Am J Trop Med Hyg.

